# Validation of a questionnaire measuring the regulation of autonomic function

**DOI:** 10.1186/1472-6882-8-26

**Published:** 2008-06-05

**Authors:** M Kröz, G Feder, HB von Laue, R Zerm, M Reif, M Girke, H Matthes, C Gutenbrunner, C Heckmann

**Affiliations:** 1 Research Institute Havelhöhe (FIH) at the Havelhöhe Community Hospital, Berlin, Germany; 2 Unit of academic primary health care, Bristol University, Bristol, UK; 3 Oncology Practice, Öschelbronn, Germany; 4 Institute for Clinical Research, Berlin, Germany; 5 Institute of Balneology and Medical Climatology, Medical University Hannover, Hannover, Germany; 6 Faculty of Human Medicine, University of Witten/Herdecke, Witten, Germany

## Abstract

**Background:**

To broaden the range of outcomes that we can measure for patients undergoing treatment for oncological and other chronic conditions, we aimed to validate a questionnaire measuring self-reported autonomic regulation (aR), i.e. to characterise a subject's autonomic functioning by questions on sleeping and waking, vertigo, morningness-eveningness, thermoregulation, perspiration, bowel movements and digestion.

**Methods:**

We administered the questionnaire to 440 participants (♀: N = 316, ♂: N = 124): 95 patients with breast cancer, 49 with colorectal cancer, 60 with diabetes mellitus, 39 with coronary heart disease, 28 with rheumatological conditions, 32 with Hashimoto's disease, 22 with multiple morbidities and 115 healthy people. We administered the questionnaire a second time to 50.2% of the participants. External convergence criteria included the German version of the Hospital Anxiety and Depression Scale (HADS-D), a short questionnaire on morningness-eveningness, the Herdecke Quality of Life Questionnaire (HLQ) and a short version questionnaire on self-regulation.

**Results:**

A principal component analysis yielded a three dimensional 18-item inventory of aR. The subscales orthostatic-circulatory, rest/activity and digestive regulation had internal consistency (Cronbach-α: rα = 0.65 – 0.75) and test-retest reliability (rrt = 0.70 – 85). AR was negatively associated with anxiety, depression, and dysmenorrhoea but positively correlated to HLQ, self-regulation and in part to morningness (except digestive aR) (0.49 – 0.13, all p < 0.05).

**Conclusion:**

An internal validation of the long-version scale of aR yielded consistent relationships with health versus illness, quality of life and personality. Further studies are required to clarify the issues of external validity, clinical and physiological relevance.

## Background

The importance of quality of life research in relation to health care evaluation is now well established. General inventories of health-related quality of life (HRQL) have established that patients with psychosomatic symptoms can have greater impairments of HRQL and daily functioning, than severely ill patients suffering from chronic physical conditions [1,2]. Smith and colleagues' meta-analysis of 12 studies [3] measuring the relationship of physical, mental and social factors and global quality of life found that HRQL was more strongly associated with mental than with physical health status. Therefore general HRQL scales potentially underestimate physical functional loss in chronically ill people [3]. Disease specific HRQL-questionnaires are more sensitive instruments for specific condition, but outcomes cannot be compared across conditions. We aimed to develop a questionnaire that measures physical symptoms related to autonomic function across a range of chronic conditions and that was sensitive enough to capture both somatic functions and somatic functional loss [4]. Physiologically measured circadian rhythm markers have been shown to be relevant for recording health [5] and illness [6]. In the case of hypertension in diabetic patients, nocturnal non-dipping correlated with increased mortality rates [7]. Reduced heart rate variability (HRV) is a risk factor for increased mortality in various chronic conditions illnesses including diabetes and coronary heart disease [8]. A loss of nocturnal cardio-respiratory coordination was seen in patients with diabetes mellitus (types 1 and 2) and with breast cancer, where the grade of loss was dependent on the severity of the illness [9]. It was also shown that the rest/activity rhythm was relevant in the prognosis for metastasised colorectal cancer [10]. Affective and autonomic disorders have been reported in manifest and latent hypothyroidism [11].

To establish a systematic method of measuring chronobiological and autonomic function, we have developed an inventory of self reported measures of autonomic regulation. It includes questions pertaining to autonomic functions such as rest/activity rhythms, vertigo, orthostatic regulation, heat regulation, metabolism and digestion; additional questions on activity aim to record self-reported sleep duration and quality, as well as day-time functioning which could reflect the rest/activity rhythm [12]. We define autonomic regulation (aR) as the state of regulation of different autonomic functions in the rhythmic change of rest and activity. aR is influenced by constitution, gender, age and disease and in healthy people, aR is a relatively stable trait. Acute illness and chronic conditions can lead to reduced aR, this is called loss of regulation [13]. The potential relevance of these autonomic functions as determinants of health and illness was first articulated by Rudolf Steiner in the 1920s [14]. He formulated questions about autonomic functions reflecting his concept of strong or weak regulation of physical and functional systems through spiritual strength, the so-called ego-organization [14].

The relation between aR and health and personality markers can also be demonstrated, which supported Steiner's idea that a strong regulation has an impact on health and on personality markers [14,15]. High aR reflects an equilibrated functioning of autonomic nervous system and with low aR indicating the converse situation [12]. In a cross-sectional study conducted from 2000 to 2001 with the short version questionnaire, we have shown that people with a range of chronic physical condition have a poor aR [13].

The variation between individuals in autonomic tone (from predominant vagotonia to sympathicotonia) is one of the most elaborated and physiologically applicable modern approaches to constitution [16]. Psychometrically measured autonomic regulation shows clear conceptual convergence to and correlation with physiological measurements of autonomic tone [17]. Moreover, constitution is an important component in some complementary medicine treatment models. In the context of anthroposophical medicine or homeopathy, constitution is central to diagnostic and treatment decisions [15,18,19]. For example calcium carbonicum can be used in the treatment of night-sweats but it also represents a constitutional type [19]. Yet we do not know if the remedy reduces night-sweats more effectively in patients with the calcium carbonicum constitution type. This type of empirical question requires valid and reliable measures of autonomic function.

Initial versions of the questionnaires testing autonomic regulation did not perform well psychometrically [18,20]. We then developed a 12 item autonomic regulation (aR) short version which had satisfactory reliability and validity [12] This version was then tested in a new sample of 408 patients with a range of medical conditions. [13] We now report the further development and re-validation of a longer (18-item) version of the aR scale which includes questions of digestive regulation with the responses of the same 408 patients in the previous analysis and an additional 32 patients with treated hypothyroidism. We aimed to validate the German version of this longer aR questionnaire and test it further with a principal component analysis.

## Methods

This multi-centre, cross-sectional study was carried out from April 2000 – November 2001. The study centres, sited in Germany, were the Havelhöhe Community Hospital (Berlin), the Öschelbronn Oncological Practice and the Wuppertal Endocrinology Practice. The participants of the study consisted of healthy volunteers and seven groups of patients. The latter were recruited consecutively among inpatients (Havelhöhe) and among outpatient consultations in the other two study centres. The conditions were: (1) histologically proven breast cancer and (2) colorectal cancer; (3) diabetes mellitus (types 1 and 2); (4) angiographically confirmed coronary artery disease (CAD); (5) clinically and immunologically confirmed rheumatological conditions (12 connective tissue disease, 11 rheumatoid arthritis, three spondylarthrosis, one polyarteritis nodosa, one polymyalgia rheumatica); (6) sonographically and immunologically confirmed (positive TPO-Ak) Hashimoto's Disease (26 (substituted) with euthyreosis and 6 with subclinical hypothyreosis) (table 1); and (7) patients with multiple conditions either with two of the study conditions (1 to 6) or one of the chronic study conditions with an additional cancer. We included these conditions to test construct validity based on hypotheses concerning aR for each condition: lowest aR was hypothesised for patients with multiple conditions, reduced aR was hypothesised in breast cancer, diabetes, and the two auto-immune diseases and no reduction in aR was hypothesised for CAD and colorectal cancer. For a detailed socio-demographic description of the sample see table 1. If a patient with an initial diagnosis of diabetes mellitus had a coronary event or new diagnosis of CAD, they were still analysed in the diabetes group. For all other cases of new co-morbidity, the patients were analysed in the multiple conditions group. The control group was recruited from the Havelhöhe Hospital staff and their relatives. 131 persons have been asked consecutively, 11 refused participation, after excluding relevant diseases the questionnaire was filled out by 115 persons. Exclusion criteria were: other severe organic conditions (with the exception of the multiple conditions group), manifest psychosis (because of potential insufficient compliance), severe immobilisation or a Karnofsky index <50%, uncontrolled pain, recent operations (<1 week prior to study recruitment) or recent chemotherapy or radiotherapy (<3 weeks prior to recruitment) to avoid the potential confounding of adverse effects of treatment and for a more robust test-retest analysis. All participants gave their written informed consent. We were not required to seek formal ethics approval for this study by our institution, although it fulfilled contemporary ethical standards for psychometric studies.

**Table 1:** Sociodemographic data of the study groups including participation refusion and exclusion rate

	**TG**	**CG**	**BC**	**CRC**	**DM (1/2)**	**CHD**	**RD**	**HD**	**MC**
Invited (n)	475	131	95	51	66	45	29	36	22
Consented (n)	452	120	95	51	60	42	28	34	22
Complete Data (n/%)	440/100	115/26.1	95/21.6	49/11.1	60/13.6 (20/40)	39/8.9	28/6.4	32/7.3	22/5.0
Women (n/%)	316/71.8	80/25.3	95/30.1	30/9.5	29/9.2 (10/19)	13/4.1	22/7.0	32/10.1	15/4.7
Men (n/%)	124/28.2	35/2.8	0/0	19/15.3	31/25.0 (10/21)	26/21.0	6/4.8	0/0	7/5.6
Age (mean)	56.8	54.0	57.1	62.0	53.7	67.3	47.7	52.1	65.1
(SD)	(13.6)	(14.2)	(9.9)	(12.2)	(14.5)	(9.3)	(16.4)	(12.6)	(9.7)
Retest (n/%)	227/51.6	59/26.0	34/15.0	26/11.4	41/18.1	22/9.7	19/8.4	17/7.5	12/5.3
Test-Retest time in weeks Mean (SD)	13.1 (7.8)								
Median (25/75% Quartils)	12.0 (6.0/19)								
**Marital Status**									
Married (n/%)	274/62.3	75/27.4	59/21.5	33/12.0	38/13.8	29/10.6	10/3.6	15/5.5	15/5.5
Single (n/%)	52/11.8	13/25.0	8/15.3	6/11.5	6/11.5	4/7.7	9/17.3	5/9.6	1/1.9
Divorced (n/%)	55/12.5	15/27.3	13/23.6	5/9.1	9/16.4	3/5.5	4/7.3	5/9.1	1/1.8
Widowed (n/%)	41/9.3	8/19.5	9/17.6	4/9.8	7/17.1	3/7.3	3/7.3	3/7.3	4/9.8
No details available (n/%)	18/4.1	4/22.2	6/33.3	1/5.6	0/0	0/0	2/11.1	4/22.2	1/56.
**Most recent profession**									
Worker (n/%)	69/15.7	6/8.7	12/17.4	12/17.4	18/26.1	9/13.0	5/7.2	3/4.3	4/5.8
Employee/civil servant (n/%)	245/55.7	72/29.4	45/18.4	22/8.9	35/14.3	25/14.2	13/5.3	19/7.7	14/5
Self employed (n/%)	48/10.9	19/39.6	8/16.7	5/10.4	3/6.2	2/4.2	6/12.5	3/6.2	2/4.2
House wife/husband (n/%)	57/12.9	12/21.1	23/40.4	10/17.5	1/1.8	3/5.3	2/3.5	4/7.0	2/3.5
Still in education (n/%)	7/1.6	3/4.3	0/0	0/0	2/28.6	0/0	2/28.6	0/0	0/0
No details available (n/%)	14/3.2	3/21.4	7/50.0	0/0	1/7.1	0/0	0/0	3/21.4	0/0

Abbreviation: Total group (TG), Control (CG), breast cancer (BC), colorectal cancer (CRC), diabetes mellitus type 1/2 (DM), coronary heart disease (CHD), rheumatic diseases (RD), Hashimoto disease (HD), multiple conditions (MC)

The study we report here includes recapitulation of the development process in order to integrate questions about gastrointestinal function. We included 32 questions at the start of the process including the 12 in the shorter aR version [12] and 20 articulated (table 2) by a panel of seven experts: 5 doctors (a gastroenterologist, two internists, a diabetologist, an endocrinologist and a specialist in oncology), a statistician and a psychometrician. We then performed reliability and factor analyses. (table 2).

**Table 2:** Diagram of the 32 items of the analysis process with symbols (^I-IV^) indicating the point of exclusion

**Non-validated items on autonomic regulation**	**Responses**		
At what age did you need your first pair of glasses?^I^			
If you tend towards vertigo, how long do your vertigo attacks usually last?^I^	seconds	minutes	hours
Do you take laxatives?^II^	frequently	occasionally	never
At what time of day do you usually have bowel movements?^II^	morning	midday	evening
You need your glasses – if at all – to see things in focus... (this question does not distinguish between farsightedness in old age and youth).^III^	nearby	no glasses necessary	in the distance
What type of flavours do you prefer at breakfast?^III^	sweet	indifferent	salty
Until 35 years of age you needed glasses to improve your vision because you were...^III^	short-sighted	no glasses necessary	far-sighted
Do you drink sweetened tea or coffee?^III^	rarely	occasionally	frequently
When taking a walk, do you usually notice anything of interest ...^III^	close by (i.e. on indifferent far away the ground etc.) (horizon etc.)		
Do you usually have bowel movements after meals?^III^	rarely	occasionally	frequently
Do you remember dreaming?^IV^	frequently	occasionally	rarely
Did you experience dental problems early in your youth^IV^	frequently	occasionally	rarely
Do you consider yourself to be skilful and dexterous?^IV^	rarely	occasionally	frequently
Can you cope with eating big meals?^IV^	hardly	reasonably well	very well

**Validated items**	**Possible answers On autonomic regulation**		
	**Low = 1**	**average = 2**	**high = 3**

Do you suffer from dizzy spells?	frequently	occasionally	never
Do you suffer from dizziness when you look down from a height?	frequently	occasionally	never
Do you suffer from dizziness when you get up in the morning?	frequently	occasionally	never
Do you suffer from dizziness when you straighten up or bend down?	frequently	occasionally	never
Do you tend to have cold or cold-sweaty hands even in the warmer months?	frequently	occasionally	rarely
Do you suffer from travel sickness (e.g. sea sickness)?	frequently	occasionally	almost/never
Do you get dizzy from circular motions (when on a roundabout, for example)?	frequently	occasionally	almost/never
**Orthostatic-circulatory regulation**			
Do you have to pull yourself together to go to work?	frequently	occasionally	rarely
Do you feel rested in the morning	rarely	occasionally	frequently
Do you have problems falling asleep?	frequently	occasionally	rarely
Do you tend to sweat?	frequently	occasionally	rarely
Do you suffer from disturbed sleep?	frequently	occasionally	rarely
At what time of the day do you feel most comfortably?	evening	in the middle of the day	morning
Do you tend to sweat at night?	frequently	occasionally	rarely
Do you tend to have stomach growling?	frequently	occasionally	rarely
**Rest/activity regulation**			
How often do you have bowel movements?	<1/day	approx. 1/day	>1/day
Do you normally have bowel movements at regular times?	rarely	occasionally	frequently
Do you suffer from constipation?	frequently	occasionally	rarely
**Digestive regulation**			
**18 item sumscale**			

(^I ^plausibility items, ^II ^answer boxes below 5% or more than 75%, ^III ^insufficient item-total/Cronbach-α, ^IV ^insufficient factor loading). 18 validated items on autonomic regulation, including the individual, possible answers, item and scale correlation with the convergence criteria aR-short version. The first twelve items are also concerning to the short version scale on aR. The left answer corresponds to low (1 point), the middle to average (2 points) and the right to high autonomic regulation (3 points).

### Convergence criteria

1) Marburg short questionnaire on chronotypology (MQC) (table 3 I) consists of 5 items relating to morningness/eveningness and is an extraction of the Horne-Ösberg Questionnaire [21] with a four or five Likert scale and a range of 4 – 24. High rating means morningness and low rating points out eveningness. It's reliability and validity are adequate: Cronbach-alpha = 0.73, test-retest reliability = 0.78) [22,23] and it is a pragmatic, short measure for characterising an individual's chronotypology.

**Table 3:** Marburg short questionnaire on chronotypology and items on the autonomic nervous system

**(I) Marburg short questionnaire on chronotypology**			**Possible answers**			
At what time would you like to get up in the morning?			before 6.20 hrs, 6.20 hrs – 7.45 hrs, 7.45 hrs – 9.50 hrs, 9.50 hrs – 10.50 hrs, after 10.50 hrs			
How difficult is it for you to get up in the morning?			not difficult	rarely difficult	not easy	very difficult
In the last 14 days, how often did you go to bed late without reason and ended up feeling that you hadn't really slept long enough?			never	once	2–3 times	frequently
At what time in the evening do you usually feel tired and find it necessary to go to bed?			before 20.50 hrs, 20.50 hrs – 22.10 hrs, 22.10 hrs – 0.50 hrs, 0.50 hrs – 2.00 hrs, after 2.00 hrs			
You will have heard of the so called "morning people", who feel at their best in the morning and „evening people", who feel at their best in the evening- which of these groups would you consider yourself to belong to?			definitely a morning person, more a morning than an evening person, more an evening person than a morning person, definitely an evening person			

**(II) Questions on autonomic function, heat sensitivity, general constitution, illnesses and symptoms**			**Possible answers**			

Do you tend to sweat through your head and extremities even in the colder months?			frequently	occasionally	rarely	
Do you tend to feel cold and to shiver?			frequently	occasionally	rarely	
In the last few years, have you reacted to common colds with a temperature over 38°?			frequently	occasionally	rarely	
Do you generally tend to feel quite low?			frequently	occasionally	rarely	
Do you have any allergies?			yes	no		
Which childhood illnesses did you have?			measles, scarlet fever, whooping cough, rubella, mumps, chicken-pox			
How many days was your average menstrual cycle? a) between the ages of 21–30 : b) at present:			over 28 days	ca. 28 days	under 28 days	
How strong are/were your period pains? a) between the ages of 21–30: b) at present:			little pain	average pain	strong pain with hours spent in bed	
BMI age 21–30	BMI age 40–45	BMI today	Kg/m^2^			

2) The German version of the Hospital Anxiety and Depression Scale (HADS-D) consists of 14 items (7 for anxiety and 7 for depression) on which people rate on a four-point Likert scale (0–21 both). Higher scoring indicate more symptoms, ≥ 11 points anxiety or depression are probable, ≥ 8 – 10 possible cases, <7 no cases. The HADS is highly reliable and valid and is an extensively used scale in family medicine and internal medicine research and audit [24,25].

3) The Grossarth-Maticek short questionnaire on self-regulation is a scale with 16 items for measuring self-regulation and health-building activity with a six-point-Likert scale. The 16 items are summed and divided by 16 to obtain a total score. A higher score indicates better self-regulation. The validity and reliability of the questionnaire are good: Cronbach-alpha = 0.80 and test-retest reliability = 0.82). We used this scale because of it's conceptual congruence with aspects of autonomic regulation. [26].

4) The Herdecke Quality of Life Questionnaire (HLQ) is a HRQL questionnaire with 35 items in 6 subscales (physical problems, digestive wellbeing, motility, mental balance, social interaction) showing good validity and reliability in large German samples. Answers are structured in a five-point-Likert scale which are transformed to percent values between 0 and 100% (best possible HRQL) [27]. We administered it as one would expect convergence between aR and HQRL. Questions on autonomic state, heat sensitivity, general constitution, current and past illnesses, medication were collected by questionnaire (table 3 II) and semi-standardised interviews [12].

### Acceptability criteria

Our criterion for acceptability of a questionnaire item was that 95% or more of participants had to complete it. Items were also dropped if less than 5% or more than 75% of participants scored one. Finally, all items with item-total-correlations less than 0.10 and greater than 0.70 were excluded.

### Principal component analysis

A principal component analysis (rotation: varimax with Kaiser normalisation) was conducted with data from all participants on the acceptable items, with an optimality assessment of the two-, three-, four- and five-factor model, respectively, at a minimum factor load of r = 0.35. Only items that loaded on a factor remained in the scale.

### Internal consistency, reliability and convergence validity analyses

For both the total score and the resulting subscales, internal consistency was assessed with the Cronbach-α coefficient [28]; test-retest-reliability and pair-wise correlations between the subscales were assessed with Spearman rank correlations. We measured convergence validity (instruments measuring aspects hypothetically influencing aR) with Spearman rank correlations [29].

### Discriminant validity

We based analysis of discriminant validity on differences between each of the chronic condition groups and the healthy group with gender as a fixed factor and age as a continuous covariate, estimating each parameter's influence on the total aR-score and each of its subscales. We adjusted the p-values and confidence intervals of the seven resulting pair-wise comparisons, controlling for the aR-score, for multiple-testing with the Dunnett adjustment in order to keep the aR-scale-wise alpha error on a global level of 5%.

All analyses were performed with SPSS 13.0 and SAS 9.1.3 statistical packages.

## Results

We invited 475 people to participate in our study; 452 consented (95%) and we had complete data on 440 participants (93%). Participants consisted of a healthy control group (n = 115) and seven patient groups (n = 325) (table 1). The average age of participants was 56.8 years (SD = 13.6), with ages ranging from 18–85 years. 227 participants (51.6%) were re-tested when they re-attended an outpatients clinic or were re-admitted two weeks to six months after the initial questionnaire administration (table 1). The second test was carried out after a median of 12.0 weeks (table 1).

### Item acceptance and selection

The item acceptance was ≥ 95% for all items with the exception of "length of vertigo attack" (27.4%). From the initial 32 items (table 2) two items (having glasses and length of vertigo attack) were used as plausibility items and not as items for reliability analysis (table 2: I), two items in the completed answer boxes were below 5% and were consequently excluded (table 2: II): "laxative taken" (frequency = 3.1%) and "when do you have bowel movement?" (evening = 4.4%). Due to insufficient item-total correlation and because of an improved Cronbach-α when the item was excluded six items (table 2: III) were dropped from the questionnaire. In this first step 22 items were pre-selected.

### Principal component analysis

On the basis of these 22 items, we performed a principal component analysis. A four-principal-component model (seven, four, three and three items, respectively) fulfilled the Kaiser criterion [30] and showed an unambiguous model of an orthostatic-circulatory, digestive and a split component of sleep and daily regulation. The most convincing model was the one with three unambiguous components (table 4) as this model represented the most combination of items with highest face validity, and because only the eigenvalues of these three factors were larger than those of equivalent null models (with only random associations) which were generated for comparison by a Monte-Carlo simulation. The last four eliminated items see table 2: IV. Therefore, we chose an 18-item total score with three subscales as the most appropriate representation of the data Factor 1 (orthostatic-circulatory regulation) explains 10.2%, factor 2 (rest/activity regulation) explains 8.2% and factor 3 (digestive regulation) explains 5.9% of total variance.

**Table 4:** List of the 18 items on autonomic regulation with the relevant factor loading (r>0.35 in bold and minor co-loading in standard letters), α-if item deleted, item-subscale correlation, internal consistency (Cronbachs-α) and test-retest reliability, mean of items and subscales with standard deviation (SD)

**Items**	**Factor 1**	**Factor 2**	**Factor 3**	α**-if item deleted subscales**	**Item-subscale Correlation**	**Cronbach-α/Test-retest**	**Mean (SD)**
dizzy spells	**0.708**	0.225	-0.034	**0.661**	**0.564**		2.49 (0.60)
dizziness when looking down	**0.593**	0.117	0.075	**0.684**	**0.451**		2.18 (0.78)
dizziness when getting up in the morning	**0.634**	0.336	-0.058	**0.670**	**0.520**		2.50 (0.62)
dizziness when straighten up or bend down?	**0.675**	0.274	-0.083	**0.662**	**0.553**		2.25 (0.63)
cold or cold-sweaty hands	**0.409**	0.075	0.075	**0.735**	**0.248**		2.48 (0.75)
travel sickness	**0.505**	-0.143	0.023	**0.716**	**0.316**		2.51 (0.69)
dizzy from circular motions	**0.607**	0.063	0.091	**0.687**	**0.443**		1.88 (0.81)
**Orthostatic-circulatory regulation**						**r_α _= 0.733/r_rt _= 0.819**	16.28 (3.05)
pulling together to go to work	0.108	**0.601**	-0.097	**0.614**	**0.390**		2.39 (0.64)
rested in the morning	-0.005	**0.700**	-0.001	**0.598**	**0.440**		2.39 (0.74)
problems falling asleep	0.105	**0.548**	0.244	**0.606**	**0.408**		2.36 (0.77)
tend to sweat	0.130	**0.416**	0.037	**0.630**	**0.315**		2.14 (0.77)
suffering from disturbed sleep	0.119	**0.636**	0.132	**0.588**	**0.472**		2.18 (0.77)
time of the day feeling most comfortably	-0.072	**0.351**	-0.090	**0.677**	**0.166**		2.07 (0.89)
sweat at night	0.175	**0.447**	0.112	**0.619**	**0.359**		2.47 (0.72)
stomach growling	0.198	**0.405**	-0.080	**0.643**	**0.261**		2.30 (0.70)
**Rest/activity regulation**						**r_α _= 0.661/r_rt _= 0.767**	18.25 (3.34)
bowel movements frequency	0.016	0.005	**0.765**	**0.535**	**0.453**		2.07 (0.58)
bowel movements regularly	0.001	0.011	**0.713**	**0.608**	**0.409**		2.49 (0.72)
constipation	0.223	0.175	**0.756**	**0.480**	**0.490**		2.67 (0.60)
**Digestive regulation**						**r_α _= 0.645/r_rt _= 0.704**	7.24 (1.46)
**Total aR-scale**						**r_α _= 0.751/r_rt _= 0.851**	41.79 (5.80)

(coefficients with p < 0.05 are presented in bold letters, p > 0.05 in standard letters)

### Reliability

All 18 items were checked for total score, seven items for the subscales orthostatic-circulatory, eight items for rest/activity- and three items for digestive regulation: The Cronbach-α coefficients varied between 0.75 – 0.65, item-subscales correlation rtr = 0.57 – 0.17 and test-retest reliability rrt = 0.85 – 0.70 (table 4)

### Convergence validity

Low-level aR-scores in sum- and subscales correlated with features of chronic conditions and mental health, with the exception of the digestive regulation subscale: anxiety (A-HADS), depression (D-HADS), low general feeling, allergies and dysmenorrhoea, (r = 0.49 – 0.13, p = 0.001). High-level aR-scores in sum- and subscales correlated with the following quality of life dimensions and personality traits: physical complaint, digestive well-being, motility, mental balance, social interaction and initiative power in the HLQ as well as high self-regulation and morningness (with the exception of no correlation between the digestive aR-scale and MQC (r = 0.41 – 0.13, all other p < 0.05). High aR-scores were associated with thermoregulation, with the exception of the digestive regulation subscale: less perspiration and less feelings of cold (r = 0.33 – 0.15) (both p < 0.05). (all convergence correlations see in table 5)

**Table 5:** List of Spearman correlations between the aR sum- and subscales and the convergence criteria

	AR- sumscale	orthostatic-circulatory regulation	rest/activity regulation	digestive regulation
AR -sumscale				
orthostatic- circulatory regulation	**0.781**			
rest/activity regulation	**0.810**	**0.373**		
digestive regulation	**0.375**	**0.150**	**0.120**	
A-HADS	**0.495**	**0.341**	**0.486**	**0.133**
D-HADS	**0.349**	**0.212**	**0.368**	**0.127**
Self- regulation	**0.346**	**0.236**	**0.332**	**0.142**
MQC	**0.306**	**0.132**	**0.403**	0.020
HLQ-Physical Complaint	**0.399**	**0.244**	**0.368**	**0.204**
HLQ-Digestive Wellbeing	**0.356**	**0.267**	**0.315**	**0.191**
HLQ-Motility	**0.400**	**0.250**	**0.379**	**0.187**
HLQ-Mental Balance	**0.518**	**0.316**	**0.527**	**0.180**
HLQ-Social Interaction	**0.391**	**0.279**	**0.383**	**0.146**
HLQ-Initiative Power	**0.373**	**0.227**	**0.366**	**0.192**
General feeling	**0.358**	**0.239**	**0.367**	**0.140**
Less Allergies	**0.184**	**0.159**	**0.151**	-0.012
Less Dysmenorrhoea	**0.325**	**0.280**	**0.262**	0.195
Less Perspiration	**0.277**	**0.156**	**0.330**	0.010
Less Feeling Cold	**0.247**	**0.307**	**0.153**	0.013

(coefficients with p < 0.05 are presented in bold letters, p > 0.05 in standard letters)

### Discriminant validity

Participants in the healthy control group consistently had the highest aR-score; participants with breast cancer, diabetes (both in sum- and subscale 2), rheumatological conditions (sum- and subscale 1) and Hashimoto disease (subscale 3) had a reduced aR-score and the multiple conditions group the lowest aR-score in all scales. Men showed a higher aR-score than women (table 6, 7). Overall 92.2% of participants with chronic conditions show a loss of autonomic regulation and 30.2% of the healthy group, which may be a constitutional problem.

**Table 6:** Raw-data means and SD separated for gender and age classes with sum-scale and subscales.

**Gender**	**Age classes**	**N**	**aR 18 Mean/SD**	**Orthostatic-circulatory regulation Mean/SD**	**rest/activity regulation Mean/SD**	**Digestive regulation Mean/SD**
Women		316	41.0/5.8	15.7/3.1	17.9/3.3	7.1/1.5
Men		124	43.8/5.2	17.4/2.9	18.7/3.4	7.3/1.4
	lower third (18–40)	59	41.3/4.6	15.9/3.0	18.3/2.8	7.1/1.3
	middle third (41–62)	230	41.5/6.1	16.1/3.1	17.8/3.5	7.3/1.4
	upper third (63–85)	151	42.4/5.6	16.4/3.2	18.4/3.3	7.0/1.6

**Table 7:** Test of discriminant validity between controls and patient groups and gender with estimation of the means of aR with a linear model with the discrete factors diagnosis and gender and the steady covariate age

**Conditions of participants**	**Estimation of the means of aR Total score**	**95%-Confidence interval**	**p-value**
**No chronic condition**	**44.8**	**43.7 – 45.8**	
**Breast cancer**	**41.6**	**40.3 – 42.9**	**0.0004**
Colorectal cancer	42.5	40.8 – 44.1	0.1284
**Diabetes mellitus**	**41.1**	**39.7 – 42.4**	**0.0002**
**Rheumatological condition**	**41.5**	**39.5 – 43.6**	**0.0304**
Coronary heart disease	42.5	40.5 – 44.4	0.2645
Hashimoto disease	43.0	41.0 – 44.9	0.4827
**Multiple conditions**	**38.3**	**36.0 – 40.5**	**<.0001**
**Gender: Women Men**	**40.5 43.4**	**39.7 – 41.2 42.3 – 44.4**	**<.0001**
	**1) Orthostatic-circulatory aR**		
**No chronic condition**	**17.4**	**16.8 – 18.0**	
Breast cancer	16.7	15.9 – 17.4	0.4443
Colorectal cancer	16.5	15.5 – 17.4	0.4036
Diabetes mellitus	16.4	15.6 – 17.1	0.1840
**Rheumatological condition**	**15.7**	**14.6 – 16.8**	**0.0463**
Coronary heart disease	16.3	15.3 – 17.4	0.4640
Hashimoto disease	17.3	16.2 – 18.4	1.0000
**Multiple conditions**	**15.4**	**14.2 – 16.7**	**0.0306**
**Gender: Women Men**	**17.4**	**15.1 – 15.9 16.8 – 18.0**	**<.0001**
	**2) Rest/activity aR**		
**No chronic condition**	**19.8**	**19.1 – 20.4**	
**Breast cancer**	**17.5**	**16.7 – 18.2**	**<.0001**
Colorectal cancer	18.5	17.5 – 19.5	0.1695
**Diabetes mellitus**	**17.6**	**16.8 – 18.4**	**0.0003**
Rheumatological condition	18.4	17.2 – 19.6	0.2256
Coronary heart disease	18.7	17.5 – 19.9	0.5671
Hashimoto disease	18.9	17.8 – 20.1	0.7362
**Multiple conditions**	**16.5**	**15.2 – 17.9**	**0.0001**
Gender: Women Men	17.9 18.5	17.9 – 18.4 17.9 – 19.2	0.1406
	**3) Digestive aR**		
**No chronic condition**	**7.6**	**7.3 – 7.9**	
Breast cancer	7.5	7.1 – 7.8	0.9917
Colorectal cancer	7.6	7.1 – 8.0	1.0000
Diabetes mellitus	7.1	6.7 – 7.5	0.2094
Rheumatological condition	7.5	6.9 – 8.0	0.9990
Coronary heart disease	7.4	6.9 – 8.0	0.9969
**Hashimoto disease**	**6.8**	**6.2 – 7.3**	**0.0446**
**Multiple conditions**	**6.3**	**5.7 – 6.9**	**0.0020**
**Gender: Women Men**	**7.0 7.4**	**6.8 – 7.2 7.1 – 7.7**	**<.0001**

A Dunnett adaption has been conducted on p-values for the error of first type including lower and upper limits of confidence intervall.

## Discussion

We developed a reliable and valid instrument for characterising autonomic regulation with 18 items [29]. As the questions on the various autonomic functions are heterogenous, we conducted a factor analysis and detected the underlying subscales with satisfactory internal consistency: sleep rhythm and daily functioning (rest/activity), orthostatic-circulatory and digestive regulation. In this re-analysis we integrated in the scale items with a clear relationship to the autonomic nervous system and that showed a clear loading pattern. Items such as dental health, dreaming, being skilful, and flavour preference did not load. To highlight the relationship of the items to underlying physiological function, we changed the name of the inventory from "endogenous" to autonomic regulation.

All items show an unambiguous factor loading pattern in the whole sample (table 4) and in patients with chronic conditions. In the healthy group the factor model is more ambiguous. This could be related to the smaller number of symptoms in people without chronic conditions and the potential greater effect of factors operating as traits. The digestive regulation subscale has low correlation with the other both subscales, which reflects the relative heterogeneity of the different autonomic functions that are integrated in the whole inventory. This does not invalidate the total score and potentially increases the utility of the subscales in different clinical conditions. Most HRQL scales report the global scores and the scores for sub-scales.

Overall, we can conclude that the long version aR scale not only has increased content and face validity but improved reliability (table 4). The test-retest reliability is satisfactory to good, in spite of the heterogeneity and long test-retest interval (13.1 weeks). The test-retest reliability is consistent with the concept of character traits, although this would need to be confirmed with a repeated measures analysis over a longer period of time. Discriminant validity show significant differences (3.2 to 6.5 in total- and subscales) between healthy people and those with chronic conditions. These absolute differences correspond to 9 – 18% relative differences, which is likely to be meaningful clinically [31]. The utility of a scale cannot be based on its psychometric properties alone. We agree with Hyland that although a "scale should satisfy certain minimum criteria (satisfying Cronbach-α and test-retest-reliability and validating criteria), they do not form an essential part of choosing between scales. The best way to select...is examine the items of the scale carefully, and judge to what extent the set of items...matches the requirements of the research" [32]. Below we discuss the sub-scales of the questionnaire.

### 1) Orthostatic-circulatory regulation

Previous research has shown that "dizziness on rising", "dizziness on bending over" and "dizziness on looking down," (classed as orthostatic vertigo) are correlated [12] to "dizzy from circular motions" and to low levels of aR [12] in patients with physical illnesses. We confirmed this interaction in this analysis and can also apply this range of symptoms to travel sickness and "dizzy from circular motions" (table 4). Orthostatic intolerance has been observed in young women with high sympathetic drive and relative instability of their autonomic nervous system [33], but also in people with diabetes mellitus, breast cancer and coronary artery disease [34], auto-immune disorders (including rheumatological conditions) and chronic fatigue syndrome [35]. The correlation between "cold extremities" (stimulated α-adrenoreceptors) with high sympathetic drive [12,36] and questions on vertigo and dizziness are weak and could be regarded as signs of malaise or loss of regulation in illness. People with multiple and rheumatological conditions have the lowest score on this subscale. Tolerance of cold is moderately associated with and less perspiration is weakly associated with high-level orthostatic-circulatory regulation, which could be linked with the thermo-regulatory threshold level between perspiration and vasoconstriction. These functions are dependent on circadian rhythm and also on vigilance, personality and gender [37].

### 2) Rest/activity regulation

The variables "difficulties falling asleep", "disturbed sleep", "having had a good sleep" and "having to pull oneself together to do something" had a wide range of inter-item-correlation (r = 0.16 to 0.56) and item-total-correlation, consistent with variation found in other studies of psychometric measures and sleep monitoring [38,39]. The interaction between physical activity, ability, "having to pull oneself together" and sleep quality ("being rested") and its relationship to circadian well-being ("morningness") has also been reported by other investigators [40,41] as has its relationship with perspiration [12,36]. The physiological mechanism of the interaction of "stomach growling" to rest/activity is unclear, but a raised low frequency (LF) power when awake and lowered high frequency (HF) power during non-REM-sleep have been observed and point towards complex autonomic dysfunction in functional digestive disorders [42]. The reduced aR total score of the breast cancer group is partly determined by reduced rest/activity regulation and could therefore reflect the patients' cancer related fatigue (CRF) which affects more than 70% of these patients during chemotherapy and 34% of the patients up to ten years after remission [43]. With different CRF inventories moderate to high correlation with aR has been documented and the association between poor rest/activity regulation to CRF has face validity [44]. The reason why the aR scale did not have discriminant validity for colorectal cancer patients may be because the conventional chemotherapy regimes for colorectal cancer in 2000–2001 (5-Fluoruracil/leucovorine) had fewer adverse effects than for breast cancer, with a CRF-prevalence range from 5 to 20% [45]. In the diabetes group the reduced rest/activity regulation could be related to sleep apnoea and restless legs syndromes (RLS) which occur in about 36% and 27% respectively in type 2 diabetes [46,47]; sleep disturbances are also elevated in diabetes mellitus type 1 [48].

### 3) Digestive regulation

Our study is the first to integrate questions on gastrointestinal symptoms into a measure of aR. Migrating motor-complexes are determinants of gastrointestinal functioning. They are dependent on circadian rhythms, with a postprandial pattern maximum at breakfast time [49] with variation between individuals. [50]. This may account for the unreliability of questions about bowel movements at specific times of day. The association between functional bowel disorders and reduced heart rate variability [42] and the influence of light on gut electrophysiological function [51] is a potential area for further investigation of digestive disorders. Reduced digestive regulation in the multiple conditions group is related to the global loss of regulation in these patients. The loss of digestive aR in those patients with Hashimoto's disease could be due to 6 patients with sub-clinical (untreated) hypothyroidism.

Patients with cancer have high levels of anxiety and depression and these may have an adverse effect on HRQL and survival [52]. Depression and anxiety is also more prevalent in people with diabetes mellitus [53,54] and heart disease, and can worsen prognosis in the latter condition [55,56]. Higher self regulation in patients with cancer or coronary heart disease is associated with improved prognosis [26,55]. It is possible that improved autonomic regulation is a potential mediator through its association with reduced anxiety, depression and self-regulation.

The moderate correlation of aR to all HRQL dimensions of the HLQ is consistent with a recent study by Mormont & Waterhouse [56] who reported a correlation between a disturbed circadian rhythm with fatigue during the day (r = 0.37) and global HRQL (r = 0.34). We consider that the strong correlation between aR and mental balance, physical complaints and gastrointestinal motility supports our conceptualisation of aR and strengthens the case for measuring it. The relationship between high level aR and morningness seen in earlier studies was confirmed [12]. Otsuka has shown that an earlier acrophase of systolic and diastolic blood pressure is accompanied by positive mood and more balanced diet [57]. This in turn concurs with our concept that morningness can be viewed as a marker of health and well-being [12] and excessive eveningness (phase delay syndrome) is associated with breast cancer [58] and shows higher prevalence in sleep and psychiatric disorders such as seasonal affective disorder, depression and alcoholic consumption [59-61].

High aR scores are correlated with cardio-respiratory coordination [12] and a loss of aR has been shown in patients with chronic medical conditions in the short-version questionnaire [13].

A limitation of our study was that validation of the scale was confined to other inventories of self-reported symptoms. Polysomnography and actimeter-based studies are required to investigate the physiological correlates of the aR-scale. Another limitation was our choice of a low threshold of 0.10 in the reliability analysis, necessitated by the heterogeneous construct of autonomic regulation, before conducting a principal component analysis. A limitation that is intrinsic to the current version of the aR scale is the measuring of a combination of trait status. This may explain why the scale has a high sensitivity (92%) for detecting loss of regulation in patients with chronic conditions but a low specificity, ascribing low aR to 30% of healthy controls, probably detecting a constitutional (trait) deficit. This hybrid nature of the scale, detecting both aR trait and loss of regulation may also explain as well why the 3 factors have a cumulative variance of only 25%. Therefore the current version of the aR scale is unlikely to be sensitive to change and therefore not useful for prospective measurement of aR in patients undergoing treatment. We are currently undertaking a validation study for the development of a state questionnaire for the assessment of aR.

There is evidence that actimetrically measured rest-activity rhythms are correlated with outcomes in metastatic colorectal cancer [10]. This supports a longstanding concept that circadian rhythms and coordination of autonomic functions are relevant to health status and in people with chronic medical conditions, is associated with prognosis [62]. Autonomic imbalance or loss of aR can be seen not only as an early sign, but also as a underlying reaction to various somatic diseases [63] and could be associated with a loss of cardio-respiratory coordination. Therefore there is a compelling reason to measure autonomic function and symptomatology and to assess rest/activity rhythms and coordination with a psychometric clinical instrument especially in multimodal CAM concepts. The aR-scale has the potential to measure constitutional pattern at the three different functional levels, which could be important for detecting the response to multimodal anthroposophical and CAM treatment. In our own practice we have applied it clinically. For example we use it to screen for potential reduced heart rate variability (HRV) in patients with diabetes, in whom low aR is associated with reduced HRV [17]. We have also used the aR inventory as a screen for sleep disorders: sleep apnoea, RLS and RLS-treatment with Zincum valerianicum [64,65]. In breast cancer in addition to its use as a screening instrument for sleep disorders and cancer related fatigue [66] we employ it for a constitutional assessment in patients receiving mistletoe therapy [67].

Further studies of the clinical application of the aR inventory are required before its potential role in clinical practice is realised.

## Conclusion

Our evaluation of a long version of the aR inventory found satisfactory to good reliability and convergent validity and reasonable discriminant validity of the subscales between different chronic conditions and healthy volunteers. The physiological and clinical relevance of aR needs to be evaluated in future studies, including investigation of loss of autonomic functioning in different conditions and its potential role as a measure of prognosis in chronic conditions. Further studies will indicate if the aR inventory has the potential to detect the subtle effects of conventional and complementary therapies on a range of symptoms experienced by patients that are not captured by current measures.

## Abbreviations

AR: autonomic regulation; CAM: Complementary and Alternative Medicine; CHD: Coronary Heart Disease; CRF: Cancer Related Fatigue, HADS: Hospital Anxiety and Depression Scale, HRQL: Health related Quality of Life; HF: High Frequency of Heart Rate Variability; HLQ: Herdecke Quality of Life Questionnaire; HRV: Heart Rate Variability; LF: Low Frequency of Heart Rate Variability; MQC: Marburger Short Questionaire on Circadian Phase Position; RLS: Restless Legs Syndrome.

## Competing interests

The authors declare that they have no competing interests.

## Authors' contributions

MK participated in the study design, carried out the study, participated in statistical analysis, interpretation of results and writing the paper and submitting it for publication. GF participated in interpretation of results, writing and submitting the paper, MR participated in statistical analysis, interpretation of results and writing the paper, HBvL participated in the study design and in data collection, RZ participated in the study design and in data collection, MG participated in the study design and data collection, HM participated in the study design and writing the paper, CG participated in writing the paper, CH participated in the study design, data collection. All authors have read and approved the final manuscript.

## Pre-publication history

The pre-publication history for this paper can be accessed here:


